# Academical Proceedings, National and Foreign

**Published:** 1822-05

**Authors:** 


### ACADEMICAL PROCEEDINGS,

### NATIONAL AND FOREIGN.

### 1. ROYAL SOCIETY OF LONDON.

THE only papers worthy of being noticed in our Journal, connected with medical science, read before this Society, since our last report, are, 1, an Essay on the Natural History of Whales, by Mr. Scoresby, jun; 2, some Observations on Diseases of the Spine, by Mr. Earle; and, 3, a continuation of Mr. Charles Bell's Speculations on the Arrangement and Distribution of certain Nerves.

Our engagements having prevented us from attending at the meetings subsequently to the Easter holydays, we are not enabled to lay before our readers an account of these various communications on the present occasion.

### 2. ATHENEUM OF MEDICINE OF PARIS.

At some of the last meetings of this Society, some very interesting statements were made, and papers read, which deserve to be mentioned.

A strong instance of the protecting power of the vaccine against small-pox was related by Dr. Kergaradec, who stated that, in a family composed of three children, one of which, a boy, had died of the natural small-pox, and the eldest girl had shown the precursory symptoms of that disease, the mother had succeeded in preventing the development of that malady in a baby of seven months old, by having her instantly vaccinated. The two children continued to live together in the same room throughout the course of the distemper, which did not fail to manifest itself in the elder girl, with all its concomitant symptoms.

A memoir was presented by Dr. Meirieu, intitled, Critical Inquiry into the supposed Faculty of the Membranes of the Ovum in assisting to dilate the Os Uteri during Labour.

A case of Frontal Tic Douloureux, cured by the Sulphate of Quinine, was reported by M. Mege.

A very interesting history of a case of Sudden Death was related by Dr. Patissier.—A gentleman, aged about sixty-five years, retired from business, and living in easy circumstances; in the full enjoyment of his health; of a fair, and rather pale, complexion; moderate in his exercise, and temperate as to diet; never having complained either of pain in the chest or palpitation at the heart; was suddenly seized, one day in October last, with a pain in the left arm, and a general *malaise*, which was attributed to rheumatism. An idea having struck the patient that he should die, a lawyer was summoned to settle his affairs. Twelve days afterwards, after a quiet breakfast, he went out to visit a few friends, to whom he complained of not being well, and of feeling as if his head were empty. On returning home, he fell down in the street, and expired.

An examination of the dead body took place twenty-four hours after the accident, in the presence of Messrs. Asselin, Marjolin, Orichel, Olivier, and Patissier. Mons. Marjolin gave it as his opinion, before the body was opened, that the patient had died of a rupture of the heart, and not of apoplexy; as, in the latter case, death is never instantaneous. On looking at the brain, considerable congestion of the sinuses of the dura mater was found; while the pia mater, as well as the cerebral mass, presented considerable infiltration. The carotids were ossified at their entrance into the cranium. The lungs were healthy. The pericardium (which appeared much distended, had a blueish colour, and presented an evident degree of fluctuation,) contained a quantity of serum and coagulated blood. The heart, being carefully washed, was found to have its natural dimensions, and to be in every respect perfect, except in the anterior portion of the left ventricle, where a rupture was observed, half an inch in length, which extended to within the cavity of the ventricle. The substance of the heart was neither softer nor thinner than usual. Externally, and not far from the laceration in question, there were a few superficial ulcerations. The mucous membrane of the stomach and bowels was somewhat redder, but decidedly not thicker, than natural.

A case of Tracheotomy, in consequence of a very severe attack of croup in a young child, was reported by M. Bricheteau, which proved unavailing, although the pseudo-membrane had been completely removed by the operation. On this occasion, M. Duparcque, who was present, stated that, in examining several children who had died of the croup, he had found that the pseudo-membrane had formed in the bronchiæ as well as in the larynx; and that, therefore, the operation of trachcotomy does not appear to be indicated in that disease. —Adjourned.

---

